# Ten simple rules for writing Dockerfiles for reproducible data science

**DOI:** 10.1371/journal.pcbi.1008316

**Published:** 2020-11-10

**Authors:** Daniel Nüst, Vanessa Sochat, Ben Marwick, Stephen J. Eglen, Tim Head, Tony Hirst, Benjamin D. Evans

**Affiliations:** 1 Institute for Geoinformatics, University of Münster, Münster, Germany; 2 Stanford Research Computing Center, Stanford University, Stanford, California, United States of America; 3 Department of Anthropology, University of Washington, Seattle, Washington, United States of America; 4 Department of Applied Mathematics and Theoretical Physics, University of Cambridge, Cambridge, Cambridgeshire, Great Britain; 5 Wild Tree Tech, Zurich, Switzerland; 6 Department of Computing and Communications, The Open University, Great Britain; 7 School of Psychological Science, University of Bristol, Bristol, Great Britain; Dassault Systemes BIOVIA, UNITED STATES

## Abstract

Computational science has been greatly improved by the use of containers for packaging software and data dependencies. In a scholarly context, the main drivers for using these containers are transparency and support of reproducibility; in turn, a workflow’s reproducibility can be greatly affected by the choices that are made with respect to building containers. In many cases, the build process for the container’s image is created from instructions provided in a Dockerfile format. In support of this approach, we present a set of rules to help researchers write understandable Dockerfiles for typical data science workflows. By following the rules in this article, researchers can create containers suitable for sharing with fellow scientists, for including in scholarly communication such as education or scientific papers, and for effective and sustainable personal workflows.

## Author summary

Computers and algorithms are ubiquitous in research. Therefore, defining the computing environment, i.e., the body of all software used directly or indirectly by a researcher, is important, because it allows other researchers to recreate the environment to understand, inspect, and reproduce an analysis. A helpful abstraction for capturing the computing environment is a container, whereby a container is created from a set of instructions in a recipe. For the most common containerisation software, Docker, this recipe is called a Dockerfile. We believe that in a scientific context, researchers should follow specific practices for writing a Dockerfile. These practices might be somewhat different from the practices of generic software developers in that researchers often need to focus on transparency and understandability rather than performance considerations. The rules presented here are intended to help researchers, especially newcomers to containerisation, leverage containers for open and effective scholarly communication and collaboration while avoiding the pitfalls that are especially irksome in a research lifecycle. The recommendations cover a deliberate approach to Dockerfile creation, formatting and style, documentation, and habits for using containers.

## Introduction

Computing infrastructure has advanced to the point where not only can we share data underlying research articles, but we can also share the code that processes these data. The sharing of code files is enabled by collaboration platforms such as GitHub or GitLab and is becoming an increasingly common practice. The sharing of the computing environment is enabled by containerisation, which allows for documenting and sharing entire workflows in a comprehensive way. Importantly, this sharing of computational assets is paramount for increasing the reproducibility of computational research. While papers based on the traditional journal article format can share extensive details about the research, computational research is often far too complicated to be effectively disseminated in this format [1]. Approaches such as containerisation are needed to support computational research, or when analysing or visualising data, because a paper’s actual contribution to knowledge includes the full computing environment that produced a result [2].

Containerisation helps provide instructions for packaging the building blocks of computer-based research (i.e., code, data, documentation, and the computing environment). Specifically, containers are built from plain text files that represent a human- and machine-readable recipe for creating the computing environment and interacting with data. By providing this recipe, authors of scientific articles greatly improve their work’s level of documentation, transparency, and reusability. This is an important part of common practice for scientific computing [3,4]. An overall goal of these practices is to ensure that both the author and others are able to reproduce and extend an analysis workflow. The containers built from these recipes are portable encapsulated snapshots of a specific computing environment that are both more lightweight and transparent than virtual machines. Such containers have been demonstrated for capturing scientific notebooks [5] and reproducible workflows [6].

**Fig 1:**
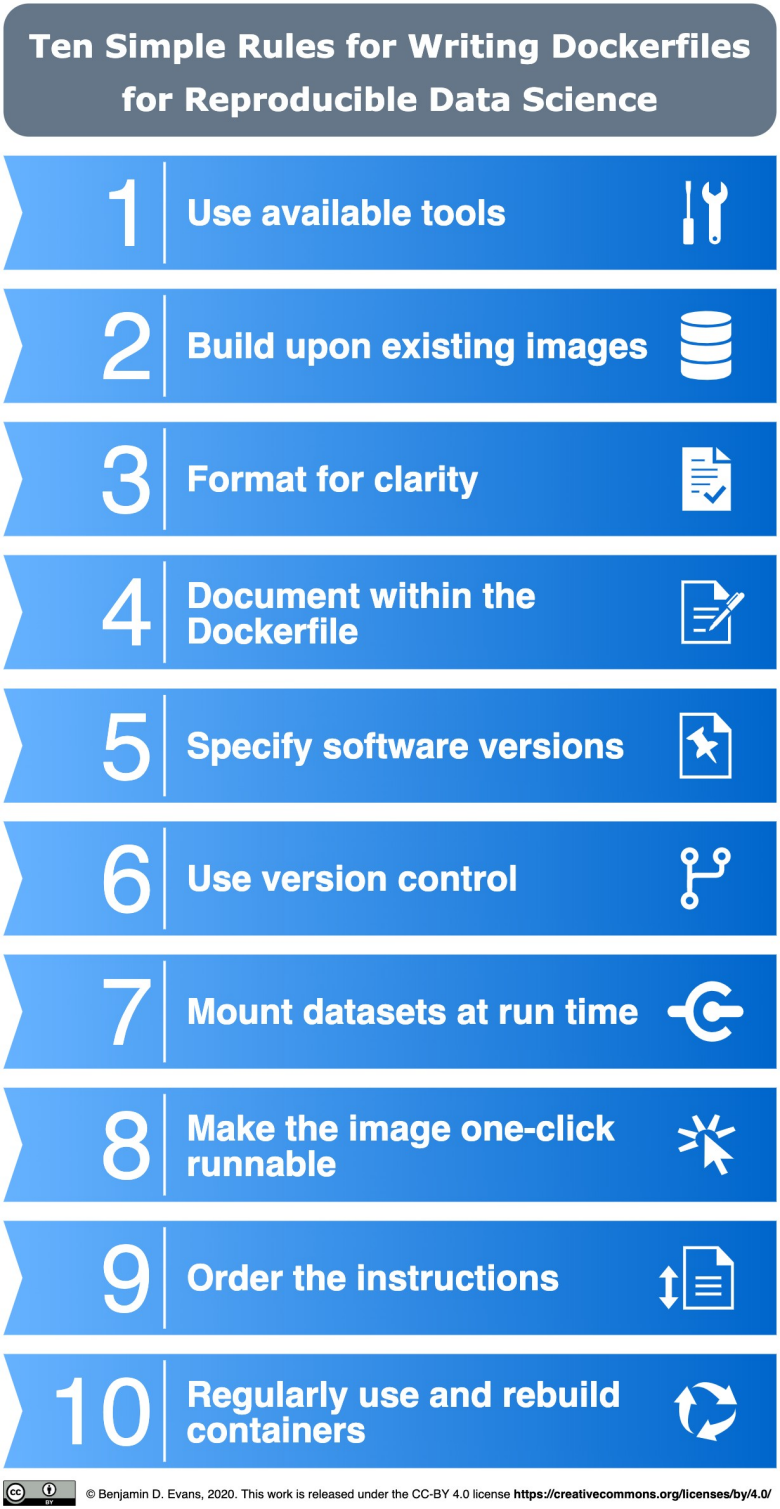
Summary of the 10 simple rules for writing Dockerfiles for reproducible data science.

While several tutorials exist on how to use containers for reproducible research ([7–11] and Gruening and colleagues [12] give very helpful recommendations for packaging reusable software in a container), there is no detailed manual for how to write the actual instructions to create the containers for computational research besides generic best practice guides [13,14]. Here, we introduce a set of recommendations for producing container configurations in the context of data science workflows using the popular Dockerfile format, summarised in Fig 1.

## Prerequisites and scope

To start with, we assume the existence of a scripted scientific workflow, i.e., you can, at least at a certain point in time, execute the full process with a fixed set of commands, for example, make prepare_data followed by Rscript analysis.R or only python3 my-workflow.py. To maximise reach, we assume that containers, which you eventually share with others, can only run open-source software; tools like Mathematica and Matlab are out of scope for this example. A workflow that does not support scripted execution is also out of scope for reproducible research, as it does not fit well with containerisation. Furthermore, workflows interacting with many petabytes of data and executed in high-performance computing (HPC) infrastructures are out of scope. Using such HPC job managers or cloud infrastructures would require a collection of “Ten Simple Rules” articles in their own right. For the HPC use case, we encourage the reader to look at Singularity [15]. For this article, we focus on workflows that typically run on single machine, e.g., a researcher’s own laptop computer or a virtual server. The reader might scope the data requirement to under a terabyte, and compute requirement to a machine with 16 cores running over the weekend.

Although it is outside the scope of this article, we point readers to docker-compose [16] in the case where one might need container orchestration for multiple applications, e.g., web servers, databases, and worker containers. A docker-compose.yml configuration file allows for defining mounts, environment variables, and exposed ports and helps users stick to “one purpose per container”, which often means one process running in the container, and to combine existing stable building blocks instead of bespoke massive containers for specific purposes.

Because “the number of unique research environments approximates the number of researchers” [17], sticking to conventions helps every researcher to understand, modify, and eventually write container recipes suitable for their needs. Even if they are not sure how the underlying technology actually works, researchers should leverage containerisation following good practices. The practices that are to be discussed in this article are strongly related to software engineering in general and research software engineering in particular, which is concerned with quality, training, and recognition of software in science [18]. We encourage you to reach out to your local or national community of research software engineers (see list of organisations) if you have questions on software development in research that go beyond the rules of this work.

While many different container technologies exist, this article focuses on Docker [19]. Docker is a highly suitable tool for reproducible research (e.g., [20]), and our observations indicate it is the most widely used container technology in academic data science. The goal of this article is to guide you as you write a Dockerfile, the file format used to create Docker container images. The rules will help you ensure that the Dockerfile allows for interactive development as well as for reaching the higher goals of reproducibility and preservation of knowledge. Such practices do not generally appear in generic containerisation tutorials, and they are rarely found in the Dockerfiles published as part of software projects that are often used as templates by novices. The differences between a helpful, stable Dockerfile and one that is misleading, prone to failure, and full of potential obstacles are not obvious, especially for researchers who do not have extensive software development experience or formal training. By committing to this article’s rules, one can ensure that their workflows are reproducible and reusable, that computing environments are understandable by others, and that researchers have the opportunity to collaborate effectively. Applying these rules should not be triggered by the publication of a finished project but should instead be weaved into day-to-day habits (cf. thoughts on openness as an afterthought by [21] and on computational reproducibility by [2]).

## Docker and Dockerfiles

Docker [19] is a container technology that has been widely adopted and is supported on many platforms, and it has become highly useful for research. Containers are distinct from virtual machines or hypervisors, as they do not emulate hardware or operating system kernels and hence do not require the same system resources. Several solutions for facilitating reproducible research are built on top of containers [17,22–25], but these solutions intentionally hide most of the complexity from the researcher.

To create Docker containers for specific workflows, we write text files that follow a particular format called Dockerfile [26]. A Dockerfile is a machine- **and** human-readable recipe for building **images**. Here, images are executable files that include the application, e.g., the programming language interpreter needed to run a workflow, and the system libraries required by an application to run. Thus, a Dockerfile consists of a sequence of instructions to copy files and install software. Each instruction adds a layer to the image, which can be cached across image builds for minimising build and download times. Once an image is built or downloaded, it is then launched as a running instance known as a **container**. The images have a main executable exposed as an “entrypoint” that is started when they are run as stateful containers. Further, containers can be modified, stopped, restarted, and purged.

**Fig 2:**
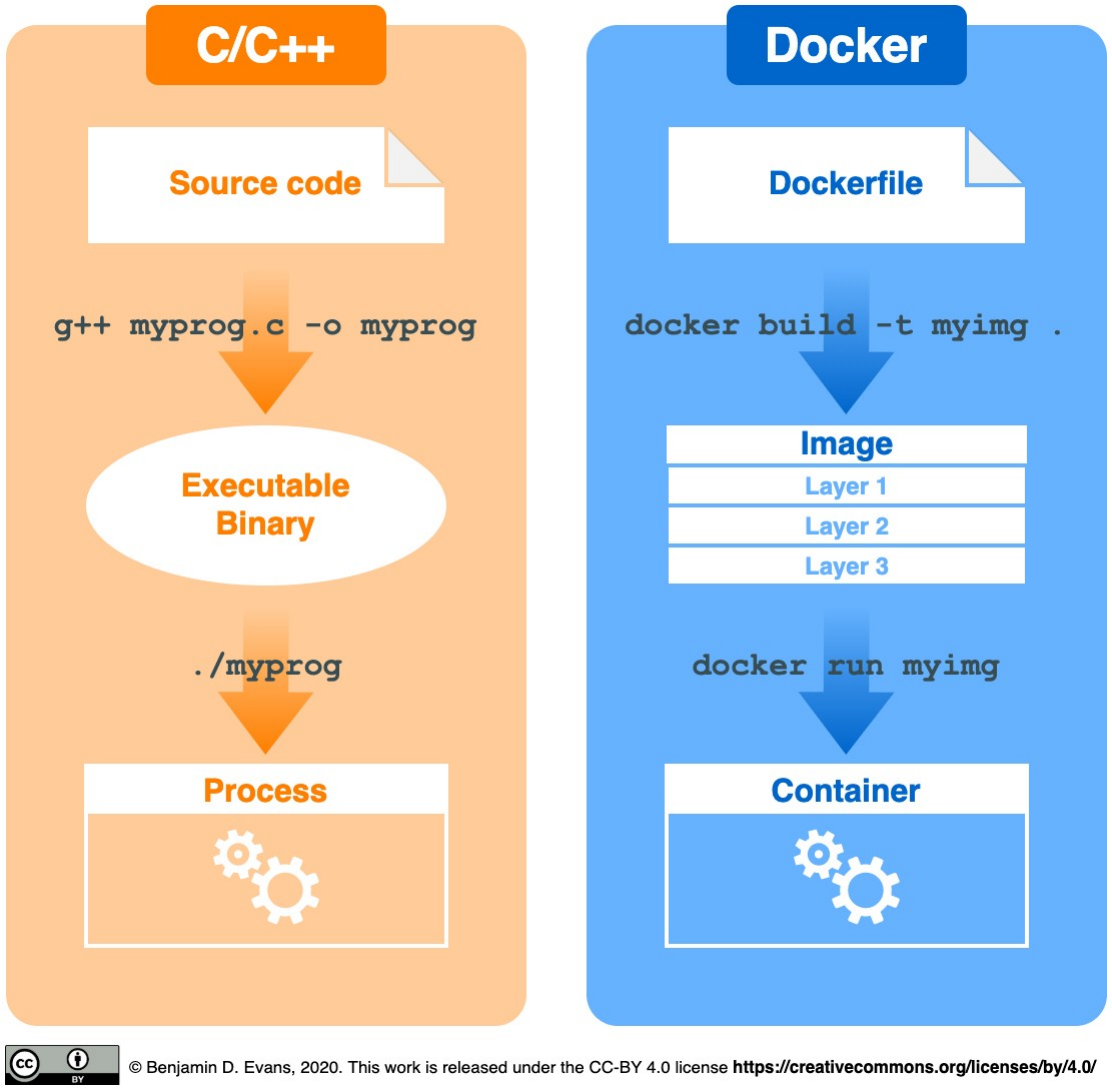
The workflow to create Docker containers by analogy. Containers begin with a Dockerfile, a recipe for building the computational environment (analogous to source code in a compiled programming language). This is used to build an image with the docker build command, analogous to compiling the source code into an executable (binary) file. Finally, the image is used to launch one or more containers with the docker run command (analogous to running an instance of the compiled binary as a process).

A visual analogy for building and running a container is provided in Fig 2. Akin to compiling source code for a programming language, creating a container also starts with a plain text file (Dockerfile), which provides instructions for building an image. Similar to using a compiled binary file to launch a program, the image is then run to create a container instance. See Listing 1 for a full Dockerfile, which we will refer to throughout this article.

While Docker was the original technology to support the Dockerfile format, other container technologies now offer support for it, including podman/buildah supported by RedHat, kaniko, img, and buildkit. The container software Singularity [15], which is optimised for scientific computing and the security needs of HPC environments, uses its own format, called the Singularity recipe, but it can also import and run Docker images. The rules here are, to some extent, transferable to Singularity recipes.

While some may argue against publishing reproducibly, e.g., due to a lack of time and incentives, a reluctance to share (cf. [28]), and the substantial technical challenges involved in maintaining software and documentation, it should become increasingly straightforward for the average researcher to provide computational environment support for their publication in the form of a Dockerfile, a pre-built Docker image, or another type of container. If a researcher can find and create containers or write a Dockerfile to address their most common use cases, then, arguably, sharing it would not make for extra work after this initial setup (cf. README.md of [29]). In fact, the Dockerfile itself represents powerful documentation to show from where data and code were derived, i.e., downloaded or installed, and, consequently, where a third party might obtain the data again.

**Listing 1.** Dockerfile full example. The Dockerfile and all other files are published in the full-demo example, see Section Examples; the image docker.io/nuest/datascidockerfiles:1.0.0 is a ready-to-use build of this example.

FROM docker.io/rocker/verse:3.6.2

### INSTALL BASE SOFTWARE #####################################################

# Install Java, needed for package rJava

RUN apt-get update && \

 apt-get install -y default-jdk && \

 rm -rf /var/lib/apt/lists/*

### INSTALL WORKFLOW TOOLS ####################################################

# Install system dependencies for R packages

RUN apt-get update && \

 apt-get install -y \

  # needed for RNetCDF, found via https://sysreqs.r-hub.io/pkg/RNetCDF

  libnetcdf-dev libudunits2-dev \

  # needed for git2r:

  libgit2-dev

# Install R packages, based on https://github.com/rocker-org/geospatial/blob/master/Dockerfile

RUN install2.r --error \

  RColorBrewer \

  RNetCDF \

  git2r \

  rJava

WORKDIR /tmp

# Install Python tools and their system dependencies

RUN apt-get update && \

 apt-get install -y python-pip && \

 rm -rf /var/lib/apt/lists/*

COPY requirements.txt requirements.txt

RUN pip install -r requirements.txt

# Download superduper image converter

RUN wget https://downloads.apache.org/pdfbox/2.0.19/pdfbox-app-2.0.19.jar

### ADD MY OWN SCRIPTS ########################################################

# Add workflow scripts

WORKDIR /work

COPY myscript.sh myscript.sh

COPY analysis.py analysis.py

COPY plots.R plots.R

# Configure workflow

ENV DATA_SIZE 42

# Uncomment the following lines to execute preprocessing tasks during build

#RUN python analysis.py

#RUN Rscript plots.R

### WORKFLOW CONTAINER FEATURE ################################################

# CMD from base image used for development, uncomment the following lines to

# have a "run workflow only" image

# CMD["./myscript.sh"]

### Usage instructions ########################################################

# Build the images with

# > docker build --tag datascidockerfiles:1.0.0.

# Run the image interactively with RStudio, open it on http://localhost/

# > docker run -it -p 80:8787 -e PASSWORD = ten --volume $(pwd)/input:/input datascidockerfiles:1.0.0

# Run the workflow:

# > docker run -it --name gwf datascidockerfiles:1.0.0 /work/myscript.sh

# Extract the data:

# > docker cp gwf:/output/ ./outputData

# Extract the figures:

# > docker cp gwf:/work/figures/ ./figures

## Rule 1: Use available tools

Rule 1 could informally be described as “Don’t bother to write a Dockerfile!”. Writing a Dockerfile from scratch can be difficult, and even experts sometimes take shortcuts. A good initial strategy is to look at tools that can help generate a Dockerfile for you. The developers of such tools have likely thought about and implemented good practices, and they may even have incorporated newer practices when reapplied at a later point in time. Therefore, the most important rule is to apply a multistep process to creating a Dockerfile for your specific use case.

First, you want to determine whether there is an existing image that you can use; if so, you want to be able to use it and add the instructions for doing so to your workflow documentation. As an example, you might be doing some kind of interactive development. For interactive development environments such as notebooks and development servers or databases, you can readily find images that come installed with all the software that you need. You can look for information about images in (a) the documentation of the software you intend to use; (b) the Docker image registry Docker Hub; or (c) the source code projects of the software being used, as many developers today rely on containers for development, testing, and teaching.

Second, if there is no suitable preexisting image for your needs, you might next look to well-maintained tools to help with Dockerfile generation. These tools can add required software packages to an existing image without you having to manually write a Dockerfile at all. “Well-maintained” not only refers to the tool’s own stability and usability but also indicates that suitable base images are used, typically from the official Docker library [30], to ensure that the container has the most recent security fixes for the operating system in question. See the next section “Tools for container generation” for details.

Third, if these tools do not meet your needs, you may want to write your own Dockerfile. In this case, follow the remaining rules.

### Tools for container generation

repo2docker [25] is a tool maintained by Project Jupyter that can help to transform a source code or data repository, e.g., GitHub, GitLab, or Zenodo, into a container. The tool relies on common configuration files for defining software dependencies and versions, and it supports a few more special files; see the supported configuration files. As an example, we might install jupyter-repo2docker and then run it against a repository with a requirements.txt file, an indication of being a Python workflow with dependencies on the Python Package Index (PyPI), using the following command:

jupyter-repo2docker https://github.com/norvig/pytudes

The resulting container image installs the dependencies listed in the requirements file, and it provides an entrypoint to run a notebook server to interact with any existing workflows in the repository. Since repo2docker is used within MyBinder.org, if you make sure your workflow is “Binder-ready”, you and others can also obtain an online workspace with a single click. However, one precaution to consider is that the default command above will create a home for the current user, meaning that the container itself would not be ideal to share; instead, any researcher interested in interacting with the code inside should run repo2docker themselves and create their own container. Because repo2docker is deterministic, the environments are the same (see Rule 5 for ensuring the same software versions).

Additional tools to assist with writing Dockerfiles include containerit [31] and dockta [32]. containerit automates the generation of a stand-alone Dockerfile for workflows in R. This utility can provide a starting point for users unfamiliar with writing a Dockerfile, or it can, together with other R packages, provide a full image creation and execution process without having to leave an R session. dockta supports multiple programming languages and configurations files, just as repo2docker does, but it attempts to create a readable Dockerfile compatible with plain Docker and to improve user experience by cleverly adjusting instructions to reduce build time. While perhaps more useful for fine-tuning, linters can also be helpful when writing Dockerfiles, by catching errors or non-recommended formulations (see Rule 10).

### Tools for templating

It is likely that over time you will work on projects and develop images that are similar in nature to each other. To avoid constantly repeating yourself, you should consider adopting a standard workflow that will give you a quick start for a new project. As an example, cookie cutter templates [33] or community templates (e.g., [34]) can provide the required structure and files (e.g., for documentation, continuous integration (CI), and licenses), for getting started. If you decide to build your own cookie cutter template, consider collaborating with your community during development of the standard to ensure it will be useful to others.

Part of your project template should be a protocol for publishing the Dockerfile and even exporting the image to a suitable location, e.g., a container registry or data repository, taking into consideration how your workflow can receive a DOI for citation. A template is preferable to your own set of base images because of the maintenance efforts the base images require. Therefore, instead of building your own independent solution, consider contributing to existing suites of images (see Rule 2) and improving these for your needs.

For any tool that you use, be sure to look at documentation for usage and configuration options, and look for options to add metadata (e.g., labels; see Rule 4).

## Rule 2: Build upon existing images

Many pre-built community and developer contributed Docker images are publically available for anyone to pull, run and extend, without having to replicate the image construction process. However, a good understanding of how **base images** and **image tags** work is crucial, as the image and tag that you choose has important implications for your derived images and containers. It is good practice to use base images that are maintained by the Docker library, so called “official images” [35], which benefit from a review for best practices and vulnerability scanning [13]. You can identify these images by the missing user portion of the image name, which comes before the /, e.g., r-base or python. However, these images only provide basic programming languages or very widely used software, so you will likely use images maintained by organisations or fellow researchers.

While some organisations can be trusted to update images with security fixes (see list below), for most individual accounts that provide ready-to-use images, it is likely that these will not be updated regularly. Further, it’s even possible that an image or a Dockerfile could disappear, or an image could be published with malicious intent (though we have not heard of any such case in academia). Therefore, for security, transparency, and reproducibility, you should only use images where you have access to the Dockerfile. In case a repository goes away, we suggest that you save a copy of the Dockerfile within your project (see Rule 7).

The following list is a selection of communities that produce widely used, regularly updated images, including ready-to-use images with preinstalled collections of software configured to work out of the box. Do take advantage of such images, especially for complex software environments, e.g., machine learning tool stacks, or a specific BLAS library.

- Rocker for R and RStudio images [20]
- Bioconductor Docker images for bioinformatics with R
- NeuroDebian images for neuroscience [36]
- Jupyter Docker Stacks for Notebook-based computing
- Taverna Server for running Taverna workflows

For example, here is how we would use a base image verse, which provides the popular Tidyverse suite of packages [37], with R version 3.5.2 from the rocker organisation on Docker Hub (docker.io, which is the default and can be omitted).

**FROM** docker.io/rocker/verse:3.6.2

### Use version-specific tags

Images have **tags** associated with them, and these tags have specific meanings, e.g., a semantic version indicator such as 3.7 or dev, or variants like slim that attempt to reduce image size. Tags are defined at the time of image build and appear in the image name after the : when you use an image, e.g., python:3.7. By convention a missing tag is assumed to be the word latest, which gives you the latest updates but is also a moving target for your computing environment that can break your workflow. Note that a version tag means that the tagged software is frozen, but it does not mean that the image will not change, as backwards compatible fixes (cf. semantic versioning, [38]), e.g., version 1.2.3 that fixes a security problem in version 1.2.2 or updates to an underlying system library, would be published to the parent tag 1.2.

For data science workflows, you should always rely on version-specific image tags, both for base images that you use and for images that you build yourself and then run (see usage instructions in Listing 1 for an example of the --tag parameter of docker build). When keeping different versions (tags) available, it is good practice to publish an image in an image registry. For details, we refer you to the documentation on automated builds, see Docker Hub Builds or GitLab’s Container Registry as well as CI services such as GitHub actions, or CircleCI that can help you get started. Do not docker push a locally built image, because that counteracts the considerations outlined above. If a pre-built image is provided in a public image registry, do not forget to direct the user to it in your documentation, e.g., in the README file or in an article.

## Rule 3: Format for clarity

First, it is good practice to think of the Dockerfile as a human- **and** machine-readable file. This means that you should use indentation, new lines, and comments to make your Dockerfile well documented and readable. Specifically, carefully indent commands and their arguments to make clear what belongs together, especially when connecting multiple commands in a RUN instruction with &&. Use \ at the end of a line to break a single command into multiple lines. This will ensure that no single line gets too long to comfortably read. Content spread across more and shorter lines also improves readability of changes in version control systems. Further, use long versions of parameters for readability (e.g.,--input instead of -i). When you need to change a directory, use WORKDIR, because it not only creates the directory if it does not exist but also persists the change across multiple RUN instructions.

Second, clarity of the steps within a Dockerfile is most important, and if it requires verbosity or adds to the final image size, that is an acceptable trade-off. For example, if your container uses a script to run a complex install routine, instead of removing it from the container upon completion (a practice commonly seen in production Dockerfiles aiming at small image sizes, cf. [12]), you should keep the script in the container for a future user to inspect; the script size is negligible compared to the image size. One common pattern you will encounter is a single and very lengthy RUN instruction chaining multiple commands to install software and clean up afterwards. For example (a) the instruction updates the database of available packages, installs a piece of software from a package repository, and purges the cache of the package manager; or (b) the instruction downloads a software’s source archive, unpacks it, builds and installs the software, and then removes the downloaded archive and all temporary files. Although this pattern creates instructions that may be hard to read, it is very common and can even increase clarity within the image file system because installation and build artifacts are gone. In general, if your container is mostly software dependencies, you should not need to worry about image size because (a) your data is likely to have much larger storage requirements; and (b) transparency and inspectability outweigh storage concerns in data science. If you really need to reduce the size, you may look into using multiple containers (cf. [12]) or multistage builds [39].

Depending on the programming language used, your project may already contain files to manage dependencies, and you may use a package manager to control this aspect of the computing environment. This is a very good practice and helpful, though you should consider the externalisation of content to outside of the Dockerfile (see Rule 7). Often, a single long Dockerfile with sections and helpful comments can be more understandable than a collection of separate files.

Generally, aim to design the RUN instructions so that each performs one scoped action, e.g., download, compile, and install one tool. This makes the lines of your Dockerfile a well-documented recipe for the user as well as a machine. Each instruction will result in a new layer, and reasonably grouped changes increase readability of the Dockerfile and facilitate inspection of the image, e.g., with tools like dive [40]. Convoluted RUN instructions can be acceptable to reduce the number of layers, but careful layout and consistent formatting should be applied.

Although you will find Dockerfiles that use build-time variables to dynamically change parameters at build time, such a customisation option reduces clarity for data science workflows.

## Rule 4: Document within the Dockerfile

### Explain in comments

As you are writing the Dockerfile, be mindful of how other people (including future you!) will read it and why. Are your choices and commands being executed clearly, or are further comments warranted? To assist others in making sense of your Dockerfile, you can add comments that include links to online forums, code repository issues, or version control commit messages to give context for your specific decisions. For example, this Dockerfile by Kaggle does a good job of explaining the reasoning behind the contained instructions. If you copy instructions from another Dockerfile, acknowledge the source in a comment. Also, it can be helpful to include comments about commands that did not work so you do not repeat past mistakes. Further, if you find that you need to remember an undocumented step, that is an indication this step should be documented in the Dockerfile. All instructions can be grouped starting with a short comment, which also makes it easier to spot changes if your Dockerfile is managed in some version control system (see Rule 6). Listing 2 shows a selection of typical kinds of comments that are useful to include in a Dockerfile.

**Listing 2.** Partial **Dockerfile** with examples for helpful comments.

…

# apt-get install specific version, use ‘apt-cache madison <pkg>‘

# to see available versions

RUN apt-get install python3-pandas = 0.23.3+dfsg-4ubuntu1

# install required R packages; before log the used repository

# for better provenance in the build log

RUN R -e ‘getOption("repos")’ && \

 install2.r \

  fortunes \

  here

# this library must be installed from source to get version newer

# than in apt sources

RUN git clone http://url.of/repo && \

 cd repo && \

 make build && \

 make install

### Add metadata as labels

Docker automatically captures useful information in the image metadata, such as the version of Docker used for building the image. The LABEL instruction can add **custom metadata** to images. You can view all labels and other image metadata with docker inspect command. Listing 3 shows the most relevant ones for data science workflows. Labels serve as structured metadata that can be leveraged by services, e.g., https://microbadger.com/labels. For example, software versions of containerised applications (cf. [12]), licenses, and maintainer contact information are commonly seen, and they are very useful if a Dockerfile is discovered out of context. Regarding licensing information, this should include the license of your own code and could point to a LICENSE file within the image (cf. [12]). While you can add arbitrarily complex information with labels, for data science scenarios the user-facing documentation is much more important. Relevant metadata that might be utilised with future tools include global identifiers such as ORCID identifiers, DOIs of the research compendium (cf. https://research-compendium.science), e.g., reserved on Zenodo, or a funding agency’s grant number. You can use the ARG instruction to pass variables at build time, for example, to pass values into labels, such as the current date or version control revision. However, a script or Makefile might be required so that you do not forget that you set the argument, or how you set it (see Rule 10).

The Open Container Initiative (OCI) Image Format Specification provides some common label keys (see the “Annotations” section in [41]) to help standardise field names across container tools, as shown below. Some keys hold specific content, e.g., org.opencontainers.image.documentation is a URL as character string pointing to documentation on the image, and org.opencontainers.image.licenses is the SPDX license identifier. You may also commonly find labels in the deprecated org.label-schema-specification format, e.g., org.label-schema.description. However, we encourage the use of the OCI schema in all new and unlabelled projects.

**Listing 3.** Partial Dockerfile with commonly used labels; note the line breaks within the values (using the \ character), which were added to limit line length, are not preceded by a space character, as this space would appear in the value, whereas the line breaks between keys and values are separated by white space for readability.

…

LABEL maintainer = "D. Nüst <daniel.nuest@uni-muenster.de>" \

 org.opencontainers.image.authors = "Nüst (daniel.nuest@uni-muenster.de), \

Sochat, Marwick, Eglen, Head, Hirst, and Evans" \

 org.opencontainers.image.url = "\

https://github.com/nuest/ten-simple-rules-dockerfiles" \

 org.opencontainers.image.documentation = "https://nuest.github.io/\

ten-simple-rules-dockerfiles/ten-simple-rules-dockerfiles.pdf" \

 org.opencontainers.image.version = "1.0.0"

LABEL org.opencontainers.image.vendor = "Ten Simple Institute, Uni of Rules" \

 org.opencontainers.image.description = "Reproducible workflow image" \

 org.opencontainers.image.licenses = "Apache-2.0"

LABEL edu.science.data.group.project = "Find out something (Grant #123456)" \

 edu.science.data.group.name = "Data Science Lab" \

 author.orcid = "0000-0002-1825-0097"

### Define versions, parameters, and paths once

The ENV instruction in a Dockerfile allows for defining environment variables. These variables persist inside the container and can be useful, for example, for (a) setting software versions or paths and reusing them across multiple instructions to avoid mistakes; (b) specifying metadata intended to be discovered by installed libraries or software; or (c) adding binaries to the path (PATH) or library path (LD_LIBRARY_PATH). You should be careful to distinguish these environment variables from those that might vary and be required at runtime. Listing 4 shows some examples. For runtime environment variables, either to set a new variable or override one set in the container, you can use the --env parameter of docker run (see Listings 4 and 6).

**Listing 4.** Partial Dockerfile showing usage of environment variables with the ENV instruction.

…

# Define number of cores used by PowerfulAlgorithm

ENV POWER_ALG_CORES 2

# Install UsefulSoft tool in specific version from source

ENV USEFULSOFT_VERSION = 1.0.0 \

 USEFULSOFT_INSTALLDIR = /workspace/bin

RUN wget http://usesoft.url/useful_software/$USEFULSOFT_VERSION/useful-$USEFULSOFT_VERSION.zip && \

 unzip useful-$USEFULSOFT_VERSION.zip -d useful-src && \

 cd useful-src && \

 bash install.sh --target $USEFULSOFT_INSTALLDIR && \

 cd .. && \

 rm -r useful-src useful-$USEFULSOFT_VERSION.zip

# Put UsefulSoft tool on the path for subsequent instructions

ENV PATH $PATH:$USEFULSOFT_INSTALLDIR

### Usage instructions ###

# […]

# Run the image (defining the number of cores used):

# > docker run --it --env POWER_ALG_CORES 32 my_workflow

### Include usage instructions

It is often helpful to provide usage instructions, i.e., how to docker build and docker run the image, **within** the Dockerfile, either at the top or bottom where the reader is likely to find them. Such documentation is especially relevant if bind mounts, specific names, or ports are important for using the container; see, for example, the final lines of Listing 1. These instructions are not limited to docker <command> but include the usage of bespoke scripts, a Makefile, or docker-compose (see Rule 8 and Rule 10). Following a common coding aphorism, we might say “A Dockerfile you wrote three months ago may just as well have been written by someone else.” Thus, usage instructions help others, because they quickly get them running your workflow and interacting with the container in the intended way without reading all of the instructions (a “tl;dr”-kind of usage). Usage instructions also provide a de facto way of testing that your container works in a way that others can try out. The Dockerfile alongside your documentation strategy is a demonstration of your careful work habits and good intentions for transparency and computational reproducibility.

## Rule 5: Specify software versions

The reproducibility of your Dockerfile heavily depends on how well you define the versions of software to be installed in the image. The more specifically you can define them the better, because using the desired version leads to reproducible builds. The practice of specifying versions of software is called **version pinning** (e.g., on apt: https://blog.backslasher.net/my-pinning-guidelines.html). For stable workflows in a scientific context, it is generally advised to freeze the computing environment explicitly and not rely on the “current” or “latest” software, which is a moving target.

### System libraries

System library versions can largely come from the base image tag that you choose to use, e.g., ubuntu:18.04, because the operating system’s software repositories are very unlikely to introduce breaking changes but will predominantly fix errors with newer versions. However, you can also install specific versions of system packages with the respective package manager. For example, you might want to demonstrate a bug, prevent a bug in an updated version, or pin a working version if you suspect an update could lead to a problem. Generally, system libraries are more stable than software modules supporting analysis scripts, but in some cases, they can be highly relevant to your workflow. Installing from source is a useful way to install very specific versions, but it comes at the cost of longer build time and more complex instructions. Here are some examples of terminal commands that will list the currently installed versions of software on your system:

- Debian/Ubuntu: dpkg --list
- Alpine: apk -vv info|sort
- CentOS: yum list installed or rpm -qa

When you install several system libraries, it is good practice to add comments about why the dependencies are needed (see Listing 1). This way, if a piece of software is removed from the container, it will be easier to remove the system dependencies that are no longer needed, thereby reducing maintenance overhead: you will not unnecessarily fix problems with a library that is no longer needed or include long-running installations. A test provided via a HEALTHCHECK [42] can further ensure proper functioning of your container.

### Version control

Software can often be installed directly from a version controlled repository (e.g., GitHub, GitLab, or Mercurial). It’s recommended to check out a specific version, tag, or commit to ensure pinning a version for the repository. For example, here is how to clone a specific release tag (v3.6.1) of the Singularity container software:

RUN git clone -b v3.6.1 https://github.com/hpcng/singularity

In the case that you want to clone and check out a specific commit, you can use the checkout command.

RUN git clone https://github.com/hpcng/singularity && \

 cd singularity && \

 git checkout 8a92cf127a49118cab61579bb36b3d51ba5c6434 && \

 *# install steps go here \*

### Extension packages and programming language modules

If you need to install packages or dependencies for a specific language, package managers are a good option. Package managers generally provide reliable mirrors or endpoints to download software; many packages are tested before release, and, most importantly, they provide access to specific versions. Most package managers have a command line interface that can be used from RUN commands in your Dockerfile, along with various flavours of “freeze” commands that can output a text file listing all software packages and versions (cf. https://markwoodbridge.com/2017/03/05/jupyter-reproducible-science.html cited by [5]). The biggest risk with using package managers with respect to a Dockerfile is outsourcing configuration. As an example, here are configuration files supported by commonly used languages in scientific programming:

- Python: requirements.txt (pip tool, [43]) and environment.yml (Conda, [44])
- R: DESCRIPTION file format [45] and r (“little R”, [46])
- JavaScript: package.json of npm [47]
- Julia: Project.toml and Manifest.toml [48].

In some cases (e.g., Conda) the package manager is also able to make decisions about what versions to install, which is likely to lead to a non-reproducible build. For this reason, it is necessary to pin the dependency versions. In the case of having few packages, it may be simplest to write the install steps and versions directly into the Dockerfile (also for clarity, see Rule 3):

RUN pip install \

geopy = = 1.20.0 \

uszipcode = = 0.2.2

Alternatively, versions may be specified in a separate dependency file (e.g., requirements.txt or environment.yml) and COPYied to the image for installation:

COPY requirements.txt.

RUN pip install -r requirements.txt

This modularisation may reduce readability, but provides more flexibility in facilitating different ways of building a reproducible environment, provided the dependency file is under version control in the same repository (see Rule 6). You can also use package managers to install software from source code COPYied into the image (see Rule 7). Finally, you can use many package managers to install software from source obtained from external code management repositories, e.g., installing a tool from a specific version tag or commit hash. Be aware of the risk that such installations may later fail, especially when the external repositories are out of your control. However, these concerns can be mitigated by running the installation command with the full URL (including the specific version tag or commit hash), which is helpful in troubleshooting if problems arise. The version pinning capabilities of these file formats and package managers are described in their respective documentation.

As a final note on software installation, you should be aware of the USER instruction in a Dockerfile and how your base image might change the user for particular instructions, restricting which commands can be run within the container. It is common to use images with the default user root, which is required for installing system dependencies. However, you may encounter base images running as a non-root user (e.g., in the Jupyter and Rocker image stacks) in order to avoid permission problems when mounting files into the container, especially for “output” files (see Rule 7). We recommend ensuring that the image works without specifying any users, and, if your image deviates from that, we suggest you document it precisely.

## Rule 6: Use version control

As plain text files, Dockerfiles are well suited for use with version control systems. Including a Dockerfile alongside your code and data is an effective way to consistently build your software, to show visitors to the repository how it is built and used, to solicit feedback and collaborate with your peers, and to increase the impact and sustainability of your work (cf. [49]).

Most importantly, you should publish **all** files COPYied into the image, e.g., test data or files for software installation from source (see Rule 7), in the same public repository as the Dockerfile, e.g., in a research compendium. If you prefer to edit your scripts more interactively in a running container (e.g., using Jupyter), then it may be more convenient to bind mount their directory from the host at run time, provided all changes are committed before sharing.

Online collaboration platforms (e.g., GitHub, GitLab) also make it easy to use CI services to test building and executing your image in an independent environment. CI increases stability and trust, and it allows for images to be published automatically. Automation strategies exist to build and test images for multiple platforms and software versions, even with CI. Such approaches are often used when developing popular software packages for a broad user base operating across a wide range of target platforms and environments, and they can be leveraged if you expect your workflow to fall into this category. Furthermore, the commit messages in your version-controlled repository preserve a record of all changes to the Dockerfile, and you can use the same versions in tags for both the container’s image and the git repository.

## Rule 7: Mount datasets at run time

The role of containers is to provide the computing environment, not to encapsulate (potentially very large) datasets. It is better to insert large data files from the local machine into the container at runtime, and use the image primarily for the software and dependencies. This insertion is achieved by using **bind mounts**. Mounting these files is preferable to using the ADD/COPY instructions in the Dockerfile, because files persist when the container instance or image is removed from your system, and the files are more accessible when the workspace is published. If you want to add local files to the container (and do not need ADD’s extra features), we recommend COPY because it is simpler and explicit. Volumes are useful for persisting changes across runs of a container and offer faster file I/O compared to other mounting methods (particularly useful with databases for example). However, they are less suitable for reproducibility, since these changes exist within the image (making them less in line with treating containers as ephemeral; see Rule 10) and are not so easy to access or place under version control. Unless specific features are needed, bind mounts are preferable to storage volumes since the contents are directly accessible from both the container and the host. The files can also be more easily included in the same repository.

Storing **data files** outside of the container allows handling of very large or sensitive datasets, e.g., proprietary data or private information. Do not include such data in an image! To avoid publishing sensitive data by accident, you can add the data directory to the .dockerignore file, which excludes files and directories from the build context, i.e., the set of files considered by docker build. Ignoring data files also speeds up the build in cases where there are very large files or many small files. As an exception, you should include dummy or small test datasets in the image to ensure that a container is functional without the actual dataset, e.g., for automated tests, instructions in the user manual, or peer review (see also “functional testing logic” in [12]). For all these cases, you should provide clear instructions in the README file on how to use the actual (or dummy) data, and how to obtain and mount it if it is kept outside of the image. When publishing your workspace, e.g., on Zenodo, having datasets outside of the container also makes them more accessible to others, for example, for reuse or analysis.

A mount can also be used to access **output data** from a container; this can be an extra mount or the same data directory. Alternatively, you can use the docker cp command to access files from a running or stopped container, but this requires a specific handling, e.g., naming the container when starting it or using multiple shells, which requires very detailed instructions for users.

You can use the -v/--volume or preferably --mount flags to docker run to configure bind mounts of directories or files [50], including options, as shown in the following examples. If the target path exists within the image, the bind mount will replace it for the started container. (Note, $HOME is an environment variable in UNIX systems representing the path to the current user’s home directory, e.g., /home/moby, and $(pwd) returns the current path).

# *mount directory*

docker run --mount type = bind,source = $HOME/project,target = /project mycontainer

# *mount directory as read-only*

docker run --mount type = bind,src = $HOME/project,dst = /workspace,readonly mycontainer

# *mount multple directories, one with write access relative to current path (Linux)*

docker run --mount type = bind,src = $(pwd)/article-x-supplement/data,dst = /input-data,readonly \–mount type = bind,src = $(pwd)/outputs,dst = /output-data mycontainer

How your container expects external resources to be mounted into it should be included in the example commands (see Rule 4). In these commands, you can also make sure to avoid issues with file permissions by using Docker’s --user option. For example, by default, writing a new file from inside the container will be owned by user root on your host, because that is the default user within the container.

## Rule 8: Make the image one-click runnable

Containers are very well suited for day-to-day development tasks (see also Rule 10), because they support common interactive environments for data science and software development. But they are also useful for a “headless” execution of full workflows. For example, [51] demonstrates a container for running an agent-based model with video files as outputs, and this article’s R Markdown source, which included cells with analysis code, is rendered into a PDF in a container. A workflow that does not support headless execution may even be seen as irreproducible.

These 2 usages can be configured by the Dockerfile’s author and exposed to the user based on the Dockerfile’s ENTRYPOINT and CMD instructions. An image’s main purpose is reflected by the default process and configuration, though the ENTRYPOINT and CMD can also be changed at runtime. It is considered good practice to have a combination of default entrypoint and command that meets reasonable user expectations. For example, a container known to be a workflow should execute the entrypoint to the workflow and perhaps use --help as the command to print out usage. The container entrypoint should **not** execute the workflow, as the user is likely to run the container for basic inspection, and starting an analysis as a surprise that might write files is undesired. As the maintainer of the workflow, you should write clear instructions for how to properly interact with the container, both for yourself and others. A possible weakness with using containers is that they can only provide one default entrypoint and command. However, tools, e.g., The Scientific Filesystem [52], have been developed to expose multiple entrypoints, environments, help messages, labels, and even install sequences. With plain Docker, you can override the defaults as part of the docker run command or in an extra Dockerfile using the primary image as a base, as shown in Listing 5. In any case, you should document different variants very well and potentially capture build and run commands in a Makefile [27]. If you use a Makefile, then keep it in the same repository (see Rule 7), and include instructions for its usage (see Rule 4). To support more complex configuration options, it is helpful to expose settings via a configuration file, which can be bind mounted from the host [51], via environment variables (see Rule 4 and [53]), or via wrappers using Docker, such as Kliko [54].

**Listing 5.** Workflow Dockerfile and derived “runner image” Dockerfile with file name Dockerfile.runner.

#----- File: Dockerfile --------------------------------------------------

# base image (interactive)

FROM jupyter/datascience-notebook:python-3.7.6

# Usage instructions:

# docker build --tag workflow:1.0.

# docker run workflow:1.0

#----- File: Dockerfile.runner -------------------------------------------

# interactive image

FROM workflow:1.0

ENTRYPOINT ["python"]

CMD ["/workspace/run-all.sh"]

# Usage instructions:

# docker build --tag workflow-runner:1.0 --file Dockerfile.runner.

# docker run -e ITERATIONS = 10 -e ALGORITHM = advanced \

#   --volume /tmp/results:/workspace/output_data workflow-runner:1.0

Interactive graphical interfaces, such as RStudio, Jupyter, or Visual Studio Code, can run in a container to be used across operating systems and both locally and remotely via a regular web browser. The HTML-based user interface is exposed over HTTP. Use the EXPOSE instruction to document the ports of interest for both humans and tools, because they need to be bound to the host to be accessible to the user using the docker run option -p/--publish <host port>:<container port>. The container should also print to the screen of the used ports along with any login credentials needed. For example, this is done in the last few lines of the output of running a Jupyter Notebook server locally (lines abbreviated):

docker run -p 8888:8888 jupyter/datascience-notebook:7a0c7325e470

[…]

[I 15:44:31.323 NotebookApp] The Jupyter Notebook is running at:

[I 15:44:31.323 NotebookApp] http://9027563c6465:8888/?token=6a92d [..]

[I 15:44:31.323 NotebookApp] or http://127.0.0.1:8888/?token=6a92 [..]

[I 15:44:31.323 NotebookApp] Use Control-C to stop this server and [..]

A person who is unfamiliar with Docker but wants to use your image may rely on graphical tools like ContainDS, Portainer, or the Docker Desktop Dashboard for assistance in managing containers on their machine without using the Docker CLI. Such tools will often detect exposed ports and declared volumes so as to make the user aware of them.

Interactive usage of a command-line interface is quite straightforward to access from containers, if users are familiar with this style of user interface. Running the container will provide a shell where a tool can be used and where help or error messages can assist the user. For example, complex workflows in any programming language can, with suitable pre-configuration, be triggered by running a specific script file. If your workflow can be executed via a command line client, you may use that to validate correct functionality of an image in automated builds, e.g., by using a small toy example and checking the output by checking successful responses from HTTP endpoints provided by the container, such as with an HTTP response code of 200, or by using a browser automation tool such as Selenium [55].

The following example runs a simple R command counting the lines in this article’s source file. The file path is passed as an environment variable.

**Listing 6.** Passing a parameter via environment variable; working code in example “pass-parameter-env”, see Examples.

docker run \

 --env CONFIG_PARAM = "/data/ten-simple-rules-dockerfiles.Rmd" \

 --volume $(pwd):/data \

 jupyter/datascience-notebook:7a0c7325e470 \

 R --quiet -e "l = length(readLines(Sys.getenv(‘CONFIG_PARAM’))); \

  print(paste(‘Number of lines: ‘, l))"

> l = length(readLines(Sys.getenv(‘CONFIG_PARAM’)));

> print(paste(‘Number of lines: ‘, l))

[1] "Number of lines: 568"

If there is only a regular desktop application, the host’s window manager can be connected to the container. Although this raises notable security issues, they can be addressed by using the “X11 forwarding” natively supported by Singularity [56], which can execute Docker containers, or by leveraging supporting tools such as x11docker [57]. Other alternatives include bridge containers [58] and exposing a regular desktop via the browser (e.g., for Jupyter Hub [59]). This variety of approaches renders seemingly more convenient uncontainerised environments unnecessary. Just using one’s local machine is only slightly more comfortable but much less reproducible and portable.

## Rule 9: Order the instructions

You will regularly build an image during development of your workflow. You can take advantage of **build caching** to avoid execution of time-consuming instructions, e.g., install from a remote resource or copying a file that gets cached. Therefore, you should keep instructions in order of least likely to change to most likely to change. Docker will execute the instructions in the order that they appear in the Dockerfile; when one instruction is completed, the result is cached, and the build moves to the next one. If you change something in the Dockerfile and rebuild the image, each instruction is inspected in turn. If it has not changed, the cached layer is used and the build progresses. Conversely, if the line has changed, that build step is executed afresh, and then every subsequent instruction will have to be executed in case the changed line influences a later instruction. You should regularly rebuild the image using the --no-cache option to learn about broken instructions as soon as possible (cf. Rule 10 as an aside, docker image prune --all is a good way to remove unused images, as these tend to accrue silently in your system and take up significant disk space). Such a rebuild is also a good occasion to revisit the order of instructions, e.g., if you appended an instruction at the end to save time while iteratively developing the Dockerfile, and the formatting. You can add a version tag to the image before the rebuild to make sure to keep a working environment at hand. A recommended ordering based on these considerations is as follows, and you can use comments to visually separate these sections in your file (cf. Listing 1):

1. System libraries
2. Language-specific libraries or modules
  - from repositories (i.e., binaries)
  - from source (e.g., GitHub)
3. Installation of your own software and scripts (if not mounted)
4. Copying data and configuration files (if not mounted)
5. Labels
6. Entrypoint and default command.

## Rule 10: Regularly use and rebuild containers

Using containers for research workflows requires not only technical understanding but also an awareness of risks that can be managed effectively by following a number of **good habits**, discussed in this section. While there is no firm rule, if you use a container daily, it is good practice to rebuild that container every 1 or 2 weeks; this helps identify breaking changes early and prevents multiple issues compounding on each other. At the time of publication of research results, it is good practice to save a copy of the image in a public data repository so that readers of the publication can access the resources that produced the published results.

First, it is a good habit to use your container every time you work on a project and not just as a final step during publication. If the container is the only platform you use, you can be highly confident that you have properly documented the computing environment [60]. You should prioritise this usage over others, e.g., noninteractive execution of a full workflow, because it gives you personally the highest value and does not limit your use or others’ use of your data and code at all (see Rule 8).

Second, for reproducibility, we can treat containers as transient and disposable, and even intentionally rebuild an image at regular intervals. Ideally, containers that we built years ago should rebuild seamlessly, but this is not necessarily the case, especially with rapidly changing technology relevant to machine learning and data science. Habitually deleting a container and performing a cache-less rebuild of the image (a) increases security due to updating underlying software; (b) helps to reveal issues requiring manual intervention, e.g., changes to code or configuration that are not documented in the Dockerfile but perhaps should be; and (c) allows you to more incrementally debug issues. This habit can be supported by using continuous deployment or CI strategies.

In case you need a setup or configuration for the first 2 habits, it is good practice to provide a Makefile alongside your Dockerfile, which can capture the specific commands. Furthermore, when you rebuild the image, you can take a fresh look at the Dockerfile and improve it over time, because it will be hard to apply all rules at once. Various linting tools, either on the command line [61] or as a web service [62], are available and can be integrated into your workflow.

Third, you can export the image to file and deposit it in a public data repository, where it not only becomes citable but also provides a snapshot of the actual environment you used at a specific point in time. You should include instructions for how to import and run the workflow based on the image archive, and add your own image tags using semantic versioning (see Rule 2) for clarity. Depositing the image next to other project files, i.e., data, code, and the used Dockerfile, in a public repository makes them likely to be preserved, but it is highly unlikely that over time you will be able to recreate it precisely from the accompanying Dockerfile. Publishing the image and the contained metadata therein (e.g., the Docker version used) may even allow future science historians to emulate the Docker environment. Sharing the actual image via a registry and a version-controlled Dockerfile together allows you to freely experiment and continue developing your workflow and keep the image up to date, e.g., updating versions of pinned dependencies (see Rule 5) and regular image building (see above).

Finally, for a sanity check and to foster even higher trust in the stability and documentation of your project, you can ask a colleague or community member to be your code copilot (see https://twitter.com/Code_Copilot) to interact with your workflow container on a machine of their own. You can do this shortly before submitting your reproducible workflow for peer review, so you are well positioned for the future of scholarly communication and open science, where these may be standard practices required for publication [21,63–65].

## Examples

To demonstrate the 10 rules, we maintain a collection of annotated example Dockerfiles in the examples directory of this article’s GitHub repository. The Dockerfiles were mostly discovered in public repositories and updated to adhere better to the rules; see https://github.com/nuest/ten-simple-rules-dockerfiles/tree/master/examples, archived at https://doi.org/10.5281/zenodo.3878582.

## Conclusions

In this article we have provided guidance for using Dockerfiles to create containers for use and communication in smaller-scale data science research. Reproducibility in research is an endeavour of incremental improvement and best efforts, not about achieving the perfect solution; such a solution may be not achievable for many researchers with limited resources, and its definition may change over time. Even if imperfect, the effort to create and document scientific workflows provides incredibly useful and valuable transparency for a project. We encourage researchers to follow these steps taken by their peers to use Dockerfiles to practice reproducible research, and we encourage them to change the way they communicate towards “preproducibility” [66], which values openness, transparency, and honesty to find fascinating problems and advance science. So, we ask researchers, with their best efforts and with their current knowledge, to strive to write readable Dockerfiles for functional containers that are realistic about what might break and what is unlikely to break. In a similar vein, we accept that researchers will freely break these rules if another approach makes more sense for their use case. Also, we ask that researchers not overwhelm themselves by trying to follow all the rules right away, but that they set up an iterative process to increase their computing environment’s manageability over time. Most importantly, we ask researchers to share and exchange their Dockerfiles freely and to collaborate in their communities to spread the knowledge about containers as a tool for research and scholarly collaboration and communication.
